# Le syndrome d'apert

**DOI:** 10.11604/pamj.2013.14.66.2178

**Published:** 2013-02-18

**Authors:** Sarra Benmiloud, Sana Chaouki, Samir Atmani, Moustapha Hida

**Affiliations:** 1 Service de pédiatrie, Hôpital Mère - Enfant, CHU Hassan II - Fès, Maroc

**Keywords:** Syndrome d'Apert, acrocéphalosyndactylie, craniosténose, exophtalmie, Apert's syndrome, acrocephalosyndactyly, craniosynostosis, exophthalmos

## Abstract

Le syndrome d'Apert est une affection congénitale rare, caractérisée par une sténose cranio-faciale associée à une syndactylie des mains et des pieds. Sa prise en charge doit être précoce et multidisciplinaire. Sa gravité réside dans la coexistence de plusieurs malformations avec un risque d'hypertension intracrânienne chronique responsable d'une cécité et d'une débilité mentale. Les auteurs rapportent une nouvelle observation à travers laquelle ils illustrent les aspects cliniques et évolutifs ainsi que les difficultés thérapeutiques de cette affection.

## Introduction

Le syndrome d'Apert est une acrocéphalosyndactilie faisant partie des sténoses crânio-faciales en rapport avec une fermeture précoce des sutures crâniennes et des altérations de la face [1]. Décrit pour la première fois en 1906, ce syndrome comporte une dysmorphie crânio-faciale, caractérisée par une brachycéphalie et un télorbitisme, associée à des syndactylies osseuses et/ou membranaires des mains et des pieds [1, 2] Il s'agit d'un syndrome polymalformatif rare de transmission autosomique dominante (1/100.000 naissances en France, et 15/100.000 naissances aux USA) touchant préférentiellement les sujets de race caucasienne, asiatique ou afro-américaine, sans prédominance de sexe [3, 4] Sa gravité réside dans la coexistence de plusieurs malformations avec un important lot de préjudices esthétiques et un risque d'hypertension intracrânienne (HTIC) chronique nécessitant une prise en charge précoce dès le bas âge.

Nous rapportons une observation typique d'un syndrome d'Apert à travers laquelle nous illustrons les aspects cliniques, évolutifs et les difficultés thérapeutiques.

## Patient et observation

A.M, garçon âgé de 10 ans, benjamin d'une fratrie quatre, sans notion de consanguinité parentale ni de cas similaires dans la famille, issu d'une grossesse menée à terme, non suivie, avec un accouchement par voie basse à domicile et un poids de naissance à 3600g. La mère était âgée de 35 ans au moment de l'accouchement et le père était âgé de 50 ans.

**Figure 1:**
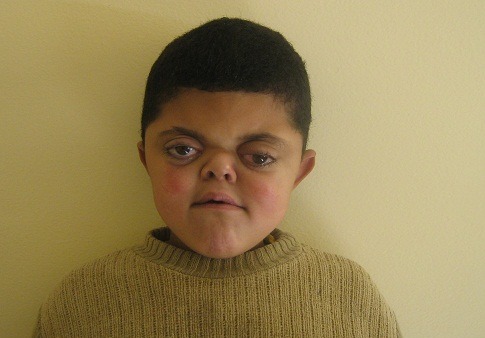
Faciès typique d'un syndrome d'Apert

**Figure 2:**
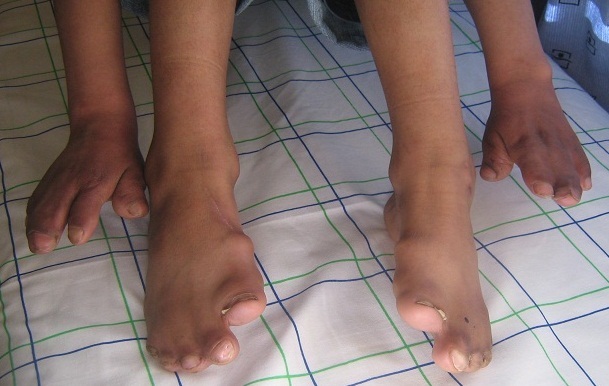
Syndactylie des deux mains et pieds avec un pouce court et large et un aspect trapu des gros orteils

L'enfant présente un retard du développement psychomoteur, des troubles du langage, un retard scolaire, des otites à répétition, une photophobie et un larmoiement évoluant depuis le bas âge. L'examen clinique à l'admission trouve une dysmorphie crânio-faciale faite d'une craniosténose type brachycéphalie, un aplatissement de l'occiput, un bombement frontal antérieur, une exophtalmie bilatérale avec lagophtalmie, un hypertélorisme, une dépression de la racine du nez avec un petit nez et une ensellure nasale ronde, un rétrognatisme, une fente palatine, une luette bifide, une malposition dentaire et des pavillons d'oreilles décollés (Figure 1). Ceci est associé à une syndactylie des mains touchant les 2^ème^, 3^ème^, 4^ème^ et 5^ème^ doigts de façon bilatérale et symétrique, un pouce court et large à son extrémité avec clinodactylie, une syndactylie des 5 orteils des pieds et des gros orteils trapus (Figure 2), une marche avec élargissement du polygone de sustentation, un rire spasmodique, un micropénis et une débilité mentale modérée.

L'examen ophtalmologique a montré des taies de la cornée prédominant du coté gauche, le segment antérieur et le fond d''il sont normaux. L'examen oto-rhino-laryngologique a objectivé des conduits auditifs rétrécis et une fente palatine incomplète. L’échocardiographie est normale. La radiographie des membres a révélé une fusion osseuse des phalanges distales des III^ème^ et IV^ème^ doigts des deux mains. Le scanner cérébral a montré une fermeture de toutes les sutures crâniennes réalisant une craniosténose avec microcrânie et une légère dilatation des ventricules latéraux. L’étude génétique n'a pu être réalisée par manque des moyens financiers des parents.

La prise en charge a consisté à la prescription de larmes artificielles pour prévenir la sécheresse oculaire et des stéristrips pour protection oculaire le soir. La neurochirurgie correctrice de la craniosténose n'a pu être réalisée car l'enfant n'a consulté qu’à l’âge de 10 ans. Par ailleurs, il est adressé en chirurgie orthopédique et réparatrice pour libération des doigts afin d'obtenir une main fonctionnelle.

## Discussion

Notre observation illustre les anomalies cliniques évocatrices du syndrome d'Apert connu aussi sous le nom d'acrocéphalosyndactylie de type 1. Il s'agit d'une brachycéphalie sévère et particulière par la persistance anormale d'une suture métopique gigantesque associée à une facio-sténose sévère. Cette dysmorphie est présente dès la naissance, donnant un tableau clinique très polyvalent qui comporte des malformations cranio-faciales en rapport avec une brachycéphalie, un aplatissement de l'occiput, un bombement frontal antérieur et une hypoplasie de l’étage moyen de la face avec un rétrécissement des loges orbitaires à l'origine d'un proptosis et parfois un exorbitisme, une exophtalmie, un hypertélorisme, un ptosis, un nez mince et pointu, un rétrognatisme, une hypoplasie des voies aériennes supérieures et de l'ethmoïde, et une fente palatine [1, 2] A ceci s'associe une syndactylie cutanée et/ou osseuse des deux mains et pieds (aspect en moufle des extrémités), de larges phalanges distales du pouce et du gros orteil et un pouce court avec clinodactylie radiale [1, 5] Cette fusion touche surtout les 2^èmes^, 3^èmes^ et 4^èmes^ doigts permettant de le différencier du syndrome de Crouzon.

Les manifestations oculaires sont très fréquentes et complexes. Il s'agit d'une exophtalmie, un hypertélorisme et un strabisme. L'altération de la fonction visuelle est la complication la plus sévère en rapport avec une kératite d'exposition, des cicatrices cornéennes, une amblyopie ou une atrophie optique [6]. Chez notre patient, l'exophtalmie avec lagophtalmie étaient responsables de kératites d'exposition avec présence de taies de la cornée. D'autres manifestations ophtalmiques sont décrites tel qu'une dysfonction de l'appareil lacrymal, une absence des muscles extra-oculaires, un albinisme oculaire, un kératocône, un glaucome congénital, une cataracte [6].

Les anomalies cardiaques et viscérales sont rencontrées dans 9,6% des cas [7]. Elles comportent une communication interventriculaire, une sténose aortique, une atrésie des choanes et de l'oesophage, une fistule trachéo-oesophagienne, une fistule anale et des anomalies génito-urinaires (hypospadias, micropénis, malposition utérine.). Les anomalies du système nerveux central comportent une agénésie du corps calleux, une hypoplasie de la substance blanche et des structures limbiques, une déformation de la selle turcique et une hydrocéphalie [8]. Une débilité mentale est décrite suite à une encéphalopathie et une hypoacousie. L'HTIC constitue le grand risque évolutif. Il s'agit d'une HTIC chronique qui évolue à bas bruit et amène de façon insidieuse à la cécité et à la débilité. D'autres anomalies peuvent être observées, tel qu'une hypopigmentation de la peau et des cheveux, des lésions acnéiformes diffuses, des malformations de la trompe d'Eustache et des otites moyennes à répétitions responsables d'une perte de l'audition [9].

Sur le plan génétique, le syndrome d'Apert est transmis sur un mode autosomique dominant, mais des cas sporadiques existent ce qui suggère le rôle de néo-mutations génétiques non héréditaires qui seraient favorisées par un âge paternel élevé [4]. Dans ce syndrome, il existe une activation du récepteur de facteurs de croissance fibroblastiques FGFR2 par mutation du gène codant son récepteur. Ces mutations seraient localisées sur les exons 5 et 7 du gène codé par l'immunoglobuline de la chaîne III [4]. Ceci a pour conséquence une augmentation du métabolisme osseux et un trouble de la synthèse osseuse. Deux types de mutations sont décrites: la S252w dans 83% des cas et la P253r dans 37% des cas [10]. La mutation S252w est plus fréquente chez les patients présentant une fente labiale tandis qu'avec la mutation P25r le degré de syndactylie est plus sévère.

L’évolution d'un enfant atteint du syndrome d'Apert dépend de l'environnement familial, d'une prise en charge précoce de la crâniosténose et de la présence ou l'absence de malformations cérébrales. Cette prise en charge nécessite une collaboration pluridisciplinaire afin d’établir un calendrier thérapeutique qui tiendra compte des différentes anomalies observées. La priorité est de lutter contre la compression du cerveau chez le nouveau-né et de gérer les problèmes cardio-respiratoires. La prise en charge de la main du syndrome d'Apert est complexe, nécessitant de nombreuses interventions avec un suivi tout au long de la croissance [5]. La neurochirurgie correctrice des crâniosténoses doit être précoce autour de l’âge de 3 mois dans le but d'enrayer ou de diminuer l'installation des troubles orbito-oculaires [8]. Les anomalies de la face nécessitent le plus souvent plusieurs interventions chirurgicales à différents âges de la vie [4]. Dans notre cas, aucune intervention sur la crâniosténose n'a pu être réalisée car l'enfant a consulté à un âge tardif.

## Conclusion

Le syndrome d'Apert est une affection rare dont le diagnostic clinique est basé sur l'association de la dysmorphie crânio-faciale et les malformations des extrémités. Sa gravité réside dans la coexistence de plusieurs malformations avec un important lot de préjudices esthétiques et un risque d'HTIC chronique responsable d'une cécité et d'une débilité mentale. L'association à des anomalies viscérales aggrave le pronostic vital et fonctionnel. Ainsi, une prise en charge précoce, patiente et de longue haleine est nécessaire pour un développement neurosensoriel sans dommage.
